# *Gelidium elegans* Extract Ameliorates Type 2 Diabetes via Regulation of MAPK and PI3K/Akt Signaling

**DOI:** 10.3390/nu10010051

**Published:** 2018-01-06

**Authors:** Jia Choi, Kui-Jin Kim, Eun-Jeong Koh, Boo-Yong Lee

**Affiliations:** Department of Food Science and Biotechnology, College of Life Science, CHA University, 463–400 Seongnam, Kyonggi, Korea; wldk3176@gmail.com (J.C.); Kuijin.Kim@gmail.com (K.-J.K.); kej763@naver.com (E.-J.K.)

**Keywords:** *Gelidium elegans* extract, type 2 diabetes, diabetic symptoms, glucose uptake, MAPK pathways, AKT

## Abstract

*Gelidium elegans*, a red alga native to the Asia Pacific region, contains biologically active polyphenols. We conducted a molecular biological study of the anti-diabetic effect of *Gelidium elegans* extract (GEE) in C57BL/KsJ-db/db mice. Mice that had been administered GEE had significantly lower body mass, water consumption, and fasting blood glucose than db/db controls. Moreover, hemoglobin A1c (HbA1c), an indicator of the glycemic status of people with diabetes, was significantly lower in mice that had been administered GEE. We also found that 200 mg/kg/day GEE upregulates the insulin signaling pathway by activating insulin receptor substrate-1 (IRS-1) and phosphoinositide 3-kinase (PI3K), and increasing the expression of glucose transporter type 4 (GLUT4). In parallel, mitogen-activated protein kinase (MAPK) activity was lower in GEE-treated groups. In summary, these findings indicate that GEE regulates glucose metabolism by activating the insulin signaling pathway and downregulating the MAPK signaling pathway.

## 1. Introduction

Diabetes mellitus (DM) is a metabolic disease that is characterized by excess glucose in the bloodstream [1]. The American Diabetes Association (ADA) reported that 12% of the annual $245 billion cost of healthcare in the USA is spent on diabetes care, and the population of diabetic individuals is rapidly growing worldwide [2]. There are two principal types of DM, characterized either by an absolute deficiency of insulin (type 1 diabetes), or resistance to the effects of insulin (type 2 diabetes; T2D) [3]. Of these, T2D is a common disorder that affects an estimated 110 million people worldwide. T2D is characterized by a disorder of insulin action, impaired glucose uptake into skeletal muscle and adipose tissue, and greater hepatic glucose output, resulting in hyperglycemia and insulin resistance [4].

Insulin signaling has been investigated for many decades, including through the mitogen-activated protein kinase (MAPK), phosphoinositol 3-kinase (PI3K)/Akt, and mechanistic target of rapamycin (mTOR) pathways [5,6,7]. Of these, the PI3K/Akt and MAPK pathways play major roles downstream of insulin receptor substrates (IRSs) [8]. The PI3K/Akt pathway has a crucial role in mediating the metabolic effects of insulin, in particular by increasing glucose uptake via the insulin-sensitive glucose transporter type 4 (GLUT4) [9]. GLUT4 is expressed in peripheral insulin-sensitive tissues, and is stored in intracellular vesicles that are mobilized to the plasma membrane immediately following insulin stimulation, thereby effecting glucose transport into cells [10,11]. Defective GLUT4 translocation is a feature of insulin resistance, which is an essential precursor of T2D [12]. Moreover, the PI3K/Akt and MAPK pathways mediate the effects of insulin on growth, the metabolism of glucose and lipids, and oxidative stress [13]. Both PI3K/Akt and signaling pathways play crucial roles in the development of diabetes. Aberrant activation of these pathways leads to abnormal glucose and lipid metabolism and oxidative stress, which are key features of diabetic pathophysiology.

There are a number of pharmacologic agents that are approved by the Food and Drug Administration (FDA) as therapies for diabetes, including metformin, rosiglitazone, and pioglitazone [14,15]. These agents help to regulate blood glucose by reducing hepatic glucose production and increasing blood glucose utilization [16,17,18]. However, although these anti-diabetic agents produce substantial reductions in blood glucose, their use is associated with a number of adverse effects, such as weight gain and gastrointestinal irritation, inducing diarrhea, nausea, and vomiting [19,20]. It is now believed that a healthy diet may be sufficient to ameliorate the symptoms of diabetes by maintaining a more normal blood glucose level [21,22,23].

Several studies report that consumption of particular phytochemicals, such as polyphenols, which are extracted from vegetables, fruits, and edible seaweeds, can reduce blood glucose both in rodent models and in human clinical trials [22,24]. Specific bioactive compounds of plant origin, including resveratrol, curcumin, and pterostilbene, have been shown to ameliorate diabetes [25,26,27]. Recent studies demonstrate that edible seaweeds may have beneficial effects in diabetes patients [28,29]. In particular, dieckol, an extract from a brown seaweed native to the Asia Pacific region, attenuates hyperglycemia through enhanced translocation of GLUT4 in peripheral tissues [29]. In addition, fucoxanthin reduces hyperglycemia in diabetic mice [30].

One example of note is *Gelidium elegans* extract (GEE), previously known as *Gelidium amansii*, an edible seaweed native to the Asia Pacific region [31]. We have described the bioactivity of GEE, including its anti-oxidant, anti-hyperglycemic, and anti-obesity effects [32,33,34]. Our previous study showed that GEE has the potential to regulate energy metabolism in high fat diet-induced obese mice [32], and in particular it may regulate glucose homeostasis [35]. The constituents of GEE are 5.1% moisture, 24.1% crude ash, 16.7% crude protein, 47.6% carbohydrate, and 8.79 mg polyphenol per g. It is a polyphenol-rich extract from an edible seaweed that may be useful for the treatment of various pathological conditions. However, although previous reports indicate that GEE might regulate blood glucose, the molecular mechanism of its anti-diabetic effects remains unclear in peripheral tissues.

We previously demonstrated that GEE affects glucose homeostasis and has an anti-hyperglycemic effect in high fat diet-induced obese mice [36]. We hypothesized that this may be achieved through the modulation of GLUT4 expression, insulin receptor substrate-1 (IRS-1)/phosphoinositide 3-kinase (PI3K), and MAPK pathways. We tested this possibility using the leptin receptor-deficient db/db mouse, a model of T2D, which exhibits a number of the typical metabolic defects seen in patients, including hyperglycemia and hyperinsulinemia [36,37], and shares many of the clinical characteristics of human T2D [37,38]. Thus, the present study investigated the effect of GEE on glucose metabolism in db/db mice by analyzing its effects on the PI3K/Akt and MAPK signaling pathways.

## 2. Materials and Methods

### 2.1. Materials

GEE was provided by NEWTREE Inc. (Kyeonggi, Korea). Its composition is shown in Table 1. Antibodies targeting GLUT4 (sc-7938), phospho-IRS-1 (sc-17196), IRS-1 (sc-559), phospho-PI3K p85α (sc-12929), phospho-c-Jun N-terminal kinases (p-JNK, sc-6254), JNK (sc-571), and glyceraldehyde 3-phosphate dehydrogenase (GAPDH, sc-25778) were purchased from Santa Cruz Biotechnology (Dallas, TX, USA). Antibodies targeting PI3K (#4255), phosphoprotein kinase B (p-Akt, #9271), Akt (#9272), phospho-p38 MAPK (p-p38 MAPK, cs-9215), p38 MAPK (#9211), phospho-p44/42 MAPK (p-ERK1/2, #4377), and ERK1/2 (#9102) were purchased from Cell Signaling Technology (Bedford, MA, USA). Metformin was obtained from the Cayman Chemical Company (Ann Arbor, MI, USA).

### 2.2. Approval of Animal Experiments

The protocols for the use of experimental animals were approved by the Institutional Animal Care and Use Committee of CHA University (IACUC 150071), and all experiments were conducted using the approved protocols and according to National Institutes of Health (NIH) guidelines.

### 2.3. Animal Husbandry and Experimental Design

This study used five-week-old male C57BL/ksJ-db/db mice and C57BL/KsJ m+/+ db mice from Central Lab Animal Inc. (Seoul, Korea). All mice were maintained in the animal facility at CHA University, Kyeonggi, Korea under a 12 h/12 h light/dark cycle at 20–24 °C and with a relative humidity of 44.5–51.8%. All experimental mice were randomly divided into five groups after a one-week adaptation period: (1) positive control group; (2) diabetic control group; (3) diabetic mice treated with metformin (140 mg/kg/day); (4) diabetic mice treated with GEE (50 mg/kg/day); (5) diabetic mice treated with GEE (200 mg/kg/day). All treatments were administered by oral gavage daily for five weeks, and all groups were fed a rodent chow diet (Zeigler Brothers, Gardners, PA, USA).

### 2.4. Body Mass, Food Intake, and Water Consumption

Body mass was measured before treatments commenced and once weekly thereafter. Food intake and water consumption were measured on the first day of treatment, and then once weekly during the experimental period. The amounts of food and water given were measured before they were supplied to each cage, and the amounts remaining the following week were measured, to calculate the weekly food intake and water consumption per unit mouse body mass.

### 2.5. Fasting Blood Glucose Measurement

Glucose was measured weekly in blood obtained from a tail vein after withholding food for 12 h during the dark period, using a glucose analyzer, GlucoDr (Allmedicus, Kyeonggi, Korea).

### 2.6. Blood Biochemistry

Mice were euthanized by overexposure to CO_2_ and cervical dislocation. Blood was collected via cardiac puncture and collected aseptically into ethylene diamine tetra-acetic acid (EDTA)-coated syringes. The blood samples were allowed to clot at room temperature for 1 h. To isolate plasma, blood was centrifuged at 13,000× *g* for 15 min at 4 °C and stored at −80 °C. Triglyceride (TG), low-density lipoprotein (LDL)-cholesterol, high-density lipoprotein (HDL)-cholesterol, and total cholesterol levels were determined using enzymatic kits (Roche, Mannheim, Germany). Glycosylated hemoglobin A1c (HbA1c) was determined by an enzymatic colorimetric method using a kit obtained from Mybiosource, San Diego, CA, USA. Insulin and C-peptide were measured using commercially available kits from Shibayagi Inc., Tokyo, Japan.

### 2.7. Organ Masses

Mice were euthanized by overexposure to CO_2_ and cervical dislocation. Subcutaneous adipose, liver, heart, lung, and skeletal muscle (thigh muscle) were removed after five weeks. Subcutaneous adipose, liver, heart, and lung were immediately weighed.

### 2.8. Western Blot Analysis

Liver and skeletal muscle samples were homogenized in Pro-prep lysis buffer (iNtRON Biotechnology, Seoul, Korea) including inhibitor cocktails 2 and 3 (Sigma, St. Louis, MO, USA), followed by centrifugation at 12,000× *g* at 4 °C for 20 min to collect the supernatant. The protein concentration of the supernatant was then determined using the Bradford method (Bio Legend, San Diego, CA, USA). Lysates (30 μg/sample) were subjected to sodium dodecyl sulfate polyacrylamide gel electrophoresis (SDS-PAGE), and then separated proteins were transferred to polyvinylidene fluoride (PVDF) membranes (Bio-Rad, Hercules, CA, USA), as previously described [39]. The membranes were then incubated with specific antibodies, and bands were detected using an enhanced chemiluminescence substrate and LAS image software (Fuji, Tokyo, Japan).

### 2.9. Statistical Analysis

Statistical analyses were performed using SPSS 12.0 (Statistical Package for Social Sciences version 12.0, Chicago, IL, USA). Body mass and fasting blood glucose were analyzed by two-way analysis of variance (ANOVA), followed by Tukey’s multiple comparisons post hoc test. The other data were analyzed by one-way ANOVA, followed by Duncan’s test. Results are expressed as mean ± standard deviation (SD). *p* < 0.05 was considered statistically significant (signified in the Figures by labels a, b, c, and d).

## 3. Results

### 3.1. Effect of GEE on Body Mass and Fasting Blood Glucose in db/db Mice

**Figure 1:**
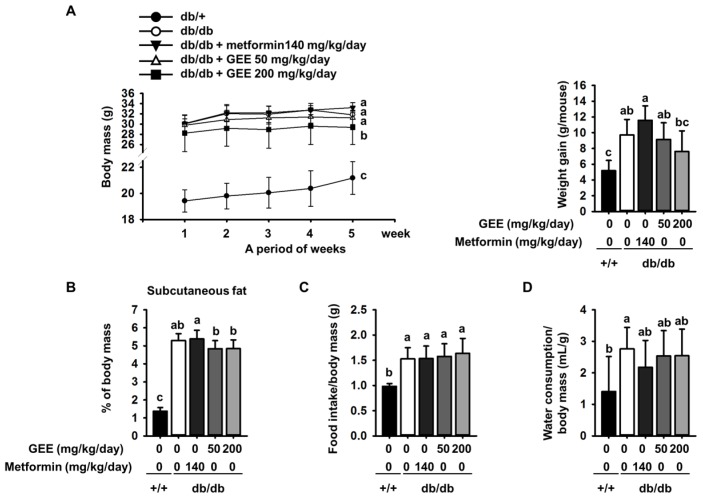
Effect of GEE on body mass (**A**); differences between time-points and treatments were analyzed by two-way ANOVA followed by Tukey’s multiple comparisons post hoc test. (*n* = 8 per group). Effect of GEE on subcutaneous fat (**B**); food intake/body mass ratio (**C**); and water consumption/body mass ratio (**D**) in db/db mice. Data were analyzed by a one-way ANOVA followed by Duncan’s test. Five-week-old mice were administered with or without GEE (50 and 200 mg/kg/day) for five weeks. Data are mean ± SD (*n* = 8 per group, ^a^ *p* < 0.05, ^b^ *p* < 0.01, ^c^ *p* < 0.001).

The body mass of each group of mice was assessed once a week during the experimental period. The initial body mass, food intake, and water consumption of the metformin, GEE 50, and GEE 200 mg/kg/day groups were not significantly different from those of the db/db control group. However, the final body masses and changes in mass among the groups were different (Figure 1A). Mice administered with 140 mg/kg/day metformin had a slightly higher final body mass (33.1 ± 1.0 g) than db/db controls (31.8 ± 0.7 g). Conversely, mice given 50 mg/kg/day GEE (31.2 ± 1.3 g) and 200 mg/kg/day GEE (29.4 ± 3.4 g) had lower final body masses than the db/db controls.

In addition, 50 and 200 mg/kg/day GEE inhibited weight gain, such that treated mice had smaller amounts of subcutaneous adipose than db/db controls (Figure 1B).

Water consumption was significantly lower in the metformin, and 50 and 200 mg/kg/day GEE, groups than in the db/db control group (Figure 1D).

Although the food intake/body mass ratio was not significantly different among the diabetic mice (Figure 1C), the db/db control group and those administered with metformin showed an increase in body mass ~4.1% greater than that shown by the db/db control group. Moreover, we observed that the masses of the liver and subcutaneous adipose depot were higher in the diabetic than the control mice at the end of the experiment (Table 2). However, 50 and 200 mg/kg/day GEE groups showed lower body, liver, and subcutaneous adipose depot mass than the db/db control group. However, the masses of the major organs (heart, lungs, spleen, and kidney) were not affected.

Finding an effective therapy for hyperglycemia is a top priority in diabetes research [40]. We therefore investigated whether GEE might have an anti-hyperglycemic effect in diabetic mice.

**Figure 2:**
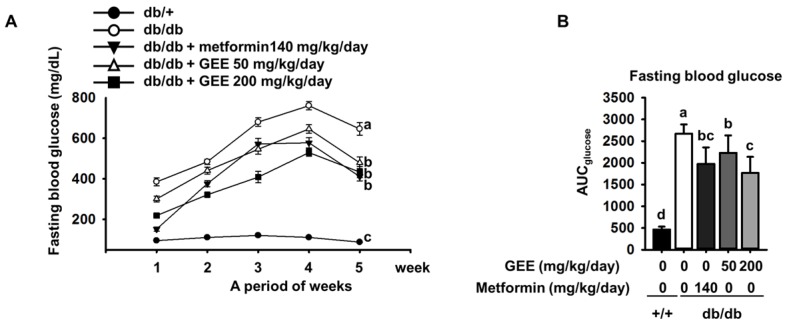
Effect of GEE on fasting blood glucose in db/db mice. Prior to measurement, five-week-old mice were fasted for 12 h during the dark period. Blood glucose levels were measured weekly. The mice were treated with or without GEE (50 and 200 mg/kg/day) (**A**). Area under the curve for glucose (AUC glucose) was calculated using the trapezoidal rule (**B**). Differences between time-points and treatments were analyzed by two-way ANOVA followed by Tukey’s multiple comparisons post hoc test. Data are mean ± SD. (*n* = 8 per group, ^a^ *p* < 0.05, ^b^ *p* < 0.01, ^c^ *p* < 0.001).

The db/db mouse demonstrates rising blood glucose with age [41]. As shown in Figure 2A, all of the diabetic groups showed high fasting glucose during the experimental period. By contrast, the fasting blood glucose level in the control group was within the normal range (105.0 ± 13.4 mg/dL).

Fasting blood glucose was significantly higher in the db/db control group than in the metformin-treated group. By contrast, the groups administered with 50 or 200 mg/kg/day GEE showed significantly lower increases in blood glucose (300.6 ± 116.9 mg/dL and 218.5 ± 100.4 mg/dL after the first week of treatment, respectively). The group that received 200 mg/kg/day GEE showed significantly lower blood glucose after one week than the db/db control group, a difference that persisted for the whole experimental period.

The area under the curve (AUC) is a useful method of comparing blood glucose between groups over a period of time [42]. We therefore calculated the AUC using the trapezoidal rule to evaluate the effect of GEE on blood glucose over the experimental period. As shown in Figure 2B, the effect of the treatment regimens on AUC glucose mirrored their effects on individual glucose measurements.

C-peptide, which is cleaved from the pro-insulin molecule prior to its release, also seems to play a role in the maintenance of normoglycemia [43]. Mice treated with 50 or 200 mg/kg/day GEE showed a lower C-peptide level than the db/db control group, and the difference in C-peptide concentration correlated with the difference in serum insulin between groups. These findings indicate that GEE suppresses insulin secretion, which may result in lower insulin resistance. Diabetes is also characterized by high serum HbA1c [44]. Therefore, we measured serum HbA1c and found that the db/db control group had significantly higher serum HbA1c levels than the positive control group (Table 3). However, the GEE groups showed much lower serum HbA1c levels, indicating that GEE may ameliorate diabetes. However, the levels of LDL, TG, and total cholesterol were not affected. These data provide preliminary evidence that GEE can ameliorate glycemic deterioration in diabetes.

### 3.2. Effect of GEE on GLUT4 Protein Expression in the Skeletal Muscle and Liver of db/db Mice

We next analyzed the skeletal muscle and liver of diabetic mice to determine the mechanism whereby GEE induced the observed effects.

**Figure 3:**
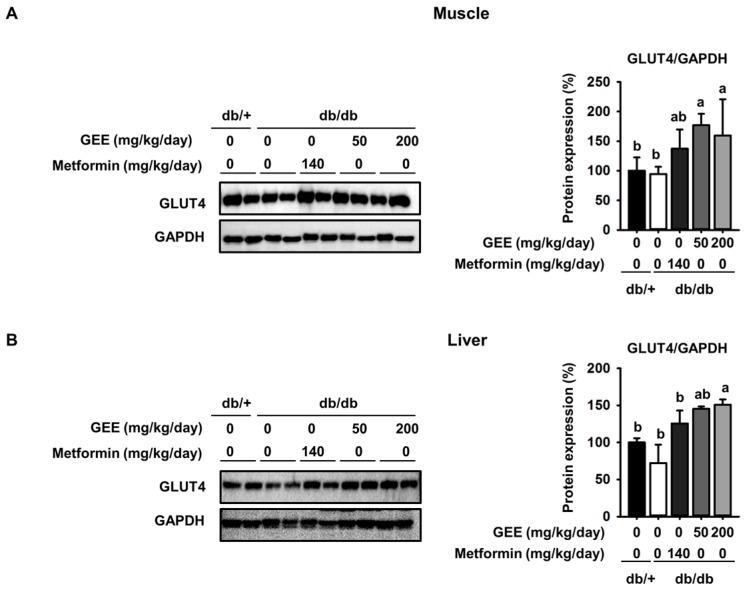
Effect of GEE on GLUT4 protein expression in the skeletal muscle and liver of db/db mice. Five-week-old mice were administered with or without GEE (50 and 200 mg/kg/day) for five weeks. GLUT4 was quantified by western blotting in the skeletal muscle (**A**) and liver (**B**). Protein expression was quantified after normalization to glyceraldehyde 3-phosphate dehydrogenase (GAPDH) using Image J software. Data were analyzed by a one-way ANOVA followed by Duncan’s test. Data are mean ± SD (*n* = 8 per group, ^a^ *p* < 0.05, ^b^ *p* < 0.01).

To clarify whether GEE could prevent the development of diabetes, we quantified a key determinant of glucose uptake, GLUT4, by western blotting in skeletal muscle and the liver. As shown in Figure 3A, metformin, and 50 and 200 mg/kg/day GEE, stimulated glucose uptake via GLUT4.

GLUT4 protein expression in the skeletal muscle was 1.7-fold higher in mice administered with 200 mg/kg/day GEE than in the db/db control group. It was also significantly higher in the liver of mice administered 200 mg/kg/day GEE (Figure 3B). The db/db control group had a lower level of GLUT4 protein, but GEE ameliorated the diabetes-induced reduction in the GLUT4 protein level. GLUT4 is the main effector of glucose uptake in peripheral insulin target tissues [45,46], and our findings indicate that GEE could regulate glucose uptake via this transporter. We next hypothesized that this effect of GEE could be mediated through the regulation of the insulin signaling pathway in skeletal muscle and the liver.

### 3.3. GEE Increases Phosphorylation of Intermediates in the PI3K/Akt Pathway in db/db Mice

Many studies report that the insulin signaling pathway, which includes IRS-1, PI3K, and Akt, is a critical regulator of GLUT4 translocation and glucose uptake [47,48,49]. To further elucidate the molecular mechanism of the effect of GEE in skeletal muscle and the liver, we used western blotting to evaluate the expression of IRS-1, PI3K, and Akt.

**Figure 4:**
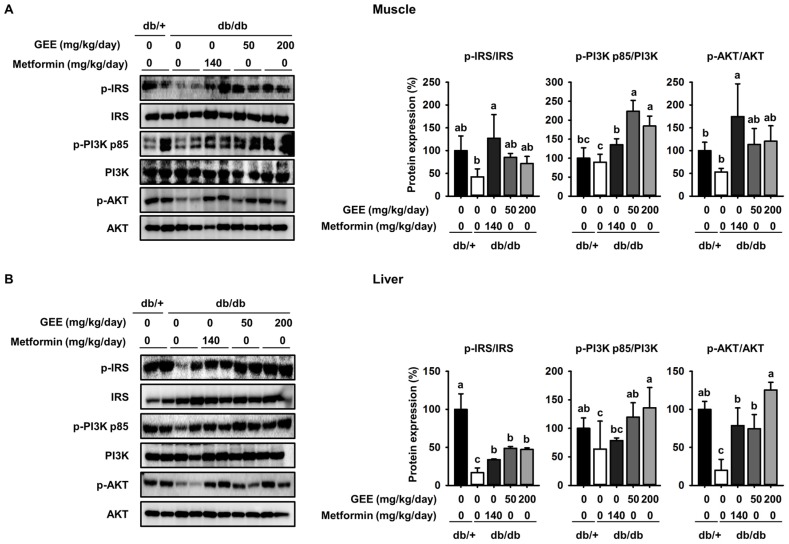
GEE increases glucose uptake via the phosphoinositide 3-kinase (PI3K)/Akt pathway in db/db mice. Five-week-old mice were administered with or without GEE (50 and 200 mg/kg/day) for five weeks. phospho-IRS-1, phospho-PI3K, and phospho-Akt were determined by western blotting in the skeletal muscle (**A**) and liver (**B**). Phosphorylation of each intermediate was quantified after normalization to total insulin receptor substrate-1 (IRS-1), phosphoinositide 3-kinase (PI3K), and Akt protein expression levels, respectively, using Image J software. Data were analyzed by a one-way ANOVA followed by Duncan’s test. Data are mean ± SD (*n* = 8 per group, ^a^ *p* < 0.05, ^b^ *p* < 0.01, ^c^ *p* < 0.001).

As shown in Figure 4A, the db/db control group had a lower level of IRS-1 phosphorylation than the metformin-treated mice, while GEE significantly increased the expression of IRS-1, PI3K, and Akt in skeletal muscle. Compared with the db/db control group, the 200 mg/kg/day GEE group showed significant increases in IRS-1, PI3K, and Akt phosphorylation of ~25%, 20%, and 38%, respectively, in skeletal muscle. We also determined the effect of GEE in the liver by western blot (Figure 3B).

Consistent with the observations in muscle, we found that 200 mg/kg/day GEE induced significant increases in hepatic IRS, PI3K, and Akt phosphorylation versus the db/db control group. These results suggest that GEE may promote glucose uptake in db/db mice via activation of the insulin signaling pathway.

### 3.4. GEE Suppresses MAPK Pathway Activation in db/db Mice

Insulin activates two main signaling pathways in its target tissues: the PI3K/Akt and the RAS-MAPK pathways. Recent studies demonstrate that MAPK pathways, including the p38 MAPK, ERK, and JNK pathways, are implicated in the control of cell development, glycolipid metabolism, inflammation, and oxidative stress, and intermediates have been shown to be phosphorylated in diabetes [50,51].

**Figure 5:**
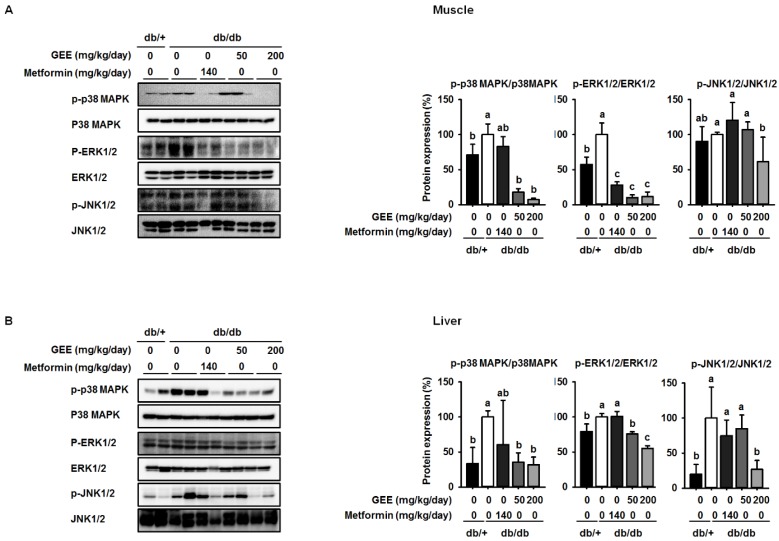
GEE suppresses mitogen-activated protein kinase (MAPK) pathway activation in db/db mice. Five-week old mice were administered with or without GEE (50 and 200 mg/kg/day) for five weeks. p-p38 MAPK, p-ERK1/2, and p-JNK1/2 were evaluated by western blotting in the skeletal muscle (**A**) and liver (**B**). Phosphorylation levels were quantified after normalization to the total p38 MAPK, ERK1/2, and JNK1/2 protein, respectively, using Image J software. Data were analyzed by a one-way ANOVA followed by Duncan’s test. Data are mean ± SD (*n* = 8 per group, ^a^ *p* < 0.05, ^b^ *p* < 0.01, ^c^ *p* < 0.001).

Western blotting was used to determine whether GEE administration decreases the expression of p38 MAPK, ERK, and JNK. The db/db control group showed higher levels of phosphorylation of p38 MAPK, ERK, and JNK than the metformin-treated mice, while mice administered 200 mg/kg/day GEE showed significantly lower phosphorylation of p38 MAPK, ERK, and JNK (by ~93%, 89%, and 39%, respectively) in the skeletal muscle than the db/db control group (Figure 5A). We also investigated the effect of GEE in liver by western blotting (Figure 4B). Consistent with the observations in muscle, 200 mg/kg/day GEE induced a significant decrease in the phosphorylation of p38 MAPK, ERK, and JNK versus db/db controls in the liver. These results suggest that GEE may ameliorate diabetes by deactivating MAPK pathways.

## 4. Discussion

Here, we show that the administration of GEE to db/db mice limits their increase in body mass and ameliorates the increase in blood glucose that characterizes their phenotype. Moreover, GEE is sufficient to ameliorate diabetic symptoms, such as extreme thirst, increased body mass, and high blood glucose, potentially through the upregulation of GLUT4 and the PI3K/Akt pathway, and the downregulation of MAPK pathway activation, in db/db mice. Our study thus demonstrates a beneficial effect of GEE on glucose uptake by the tissues of diabetic mice, which may be achieved through improved insulin sensitivity, possibly via the inhibition of MAPK pathways.

Diabetes is a metabolic disease in which the body does not appropriately process glucose, resulting in hyperglycemia [52]. In this study, fasting blood glucose and HbA1c were high in the db/db control group, confirming well-established diabetes in these mice [45]. The therapeutic effects of GEE on metabolic disorders have been reported previously. Recent studies by ourselves and others show that GEE can ameliorate hyperglycemia in high fat diet-fed mice [32]. Consistent with these previous results, we show that GEE reduces fasting blood glucose in diabetic mice.

Insulin resistance in T2D results in impaired insulin-stimulated glucose transport and metabolism in skeletal muscle and adipose, and the defective suppression of hepatic glucose output [53].

Skeletal muscle and the liver are major contributors to glucose metabolism, and GLUT4 is the principal means whereby glucose is delivered intracellularly [54,55]. Our results show that GEE administration upregulates GLUT4 protein expression in the liver and muscle of insulin resistant mice. These data suggest that GEE may lower blood glucose by enhancing glucose uptake into peripheral tissues.

The insulin signaling pathway, which includes the key mediators IRS-1, PI3K, and Akt, plays crucial cellular and molecular roles, including the synthesis and degradation of glycogen, lipid, and proteins [56,57]. Many studies show that impaired IRS-1 activation and PI3/Akt signaling suppresses glucose uptake and GLUT4 translocation [47]. Previous studies indicated that GEE can improve hyperglycemia and regulate glucose homeostasis in vivo [35]. However, these studies did not show a molecular mechanism for the effect of GEE. In the present study, GEE reversed the diabetes-induced downregulation of p-IRS, p-PI3K, and p-Akt in peripheral tissues, indicating that GEE restores insulin signaling in db/db mice.

In addition to the PI3K/Akt signaling pathway, a number of other signaling pathways have been implicated in the pathogenesis of diabetes, being involved in abnormal glucose regulation, glucolipid metabolism, and oxidative stress. These include the AMP-activated protein kinase (AMPK) pathway and MAPK pathways [58,59,60]. Furthermore, the PI3K/Akt pathway might also affect the activation of the MAPK signaling cascade [61]. To establish whether GEE has its effects on glucose homeostasis through MAPK pathways, we analyzed the expression of MAPK genes in the skeletal muscle and liver of db/db mice and found that MAPK activation, assessed by measuring the phosphorylation of p38 MAPK, ERK, and JNK, was markedly lower.

**Figure 6:**
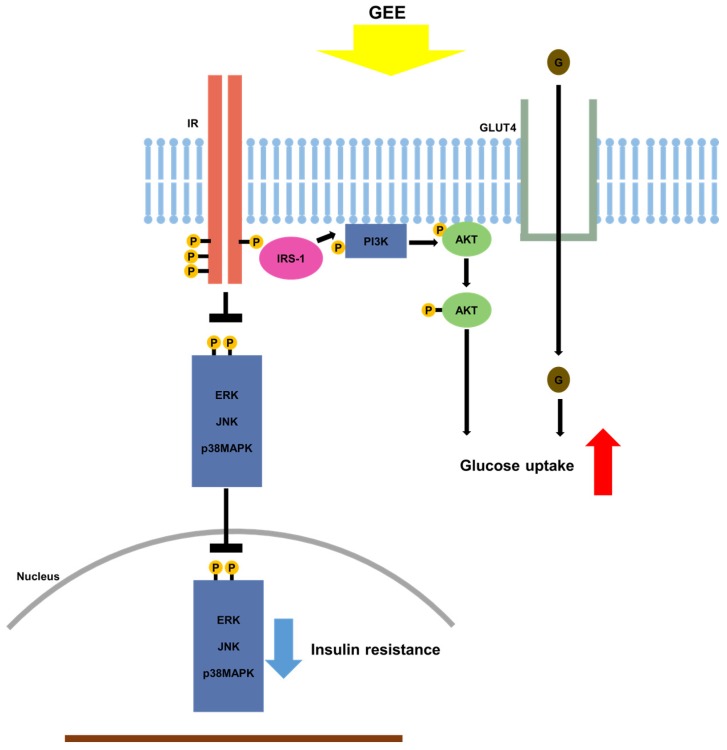
Proposed mechanism whereby GEE ameliorates type 2 diabetes through the regulation of MAPK and PI3K/Akt signaling. *Gelidium elegans* extract (GEE) stimulates glucose uptake and thus should ameliorate the clinical signs of diabetes, through activation of the phosphatidylinositol-3 kinase (p-PI3K)/protein kinase B (Akt) signaling pathway and suppression of mitogen-activated protein kinase (MAPK) signaling pathways. → indicates stimulation; ┴, inhibition; IR, insulin receptor; GLUT4, glucose transporter type 4; ERK, p44/42 MAPK; JNK; c-Jun N-terminal kinases.

Previous work demonstrated that the activation of MAPK signaling cascades may suppress the phosphorylation of IRS, leading to insulin resistance [61]. Interestingly, the activation of MAPK signaling cascades downregulates GLUT4 expression, resulting in reduced glucose transport [62]. In the present study, the activation of p38 MAPK, ERK, and JNK in the skeletal muscle and liver of db/db mice was significantly suppressed by the administration of GEE. These findings suggest that GEE may increase glucose uptake and ameliorate insulin resistance by reducing p38 MAPK, ERK, and JNK phosphorylation (Figure 6).

In many studies, body mass and subcutaneous adipose mass have been shown to be higher in diabetes [63]. In addition, a number of widely prescribed diabetes drugs have been shown to lower blood glucose by upregulating the transport of glucose into cells [64,65]. Visceral fat accumulation can contribute to this reduction in glucose uptake, poor metabolic control, and the development of diabetes complications. We previously showed that GEE-treated mice have less adipose tissue, both subcutaneous and abdominal, in association with higher uncoupling protein-1 activation [32]. Consistent with our previous results, we show here that GEE decreased body and organ mass, including that of visceral fat and liver. This might indicate that GEE promotes mitochondrial activity in brown adipose tissue, in which metabolic energy is dissipated as heat. GEE treatment may also reduce visceral fat mass, but further studies are needed to verify the involvement of altered mitochondrial activity in the anti-diabetic actions of GEE.

In summary, our study demonstrates that GEE lowers blood glucose and relieves the clinical signs of diabetes in mice, likely mediated through greater glucose uptake in peripheral tissues through the upregulation of GLUT4 and the PI3K/Akt pathway. Moreover, GEE activates IRS-1 and inhibits activation of MAPK pathways, and therefore should ameliorate insulin resistance and the clinical signs of diabetes. These data support the potential use of GEE for the treatment of diabetes.

## Figures and Tables

**Table 1:** *Gelidium elegans* extract (GEE) composition.

Nutrient	Ingredient	Content
Proximate composition (%)	Moisture	5.1
	Crude ash	24.1
	Crude protein	16.7
	Carbohydrate	47.6

**Table 2:** Effect of GEE on organ masses in db/db mice.

	Group	Organ Mass (g)				
		db/+	db/db			
Parameters		GEE 0 *	GEE 0 *	GEE 0 *	GEE 50 *	GEE 200 *
		Metformin 0 *	Metformin 0 *	Metformin 140 *	Metformin 0 *	Metformin 0 *
Subcutaneous fat		0.30 ± 0.04 ^d^	1.63 ± 0.13 ^b^	1.83 ± 0.21 ^a^	1.53 ± 0.16 ^bc^	1.45 ± 0.13 ^c^
Liver		1.08 ± 0.07 ^d^	2.13 ± 0.18 ^a^	1.89 ± 0.15 ^bc^	2.05 ± 0.15 ^ab^	1.84 ± 0.19 ^c^
Heart		0.16 ± 0.03	0.15 ± 0.03	0.16 ± 0.01	0.15 ± 0.02	0.15 ± 0.02
Lung		0.18 ± 0.03	0.19 ± 0.04	0.19 ± 0.03	0.19 ± 0.03	0.19 ± 0.03

* (mg/kg/day), (*n* = 8 per group, ^a^ *p* < 0.05, ^b^ *p* < 0.01, ^c^ *p* < 0.001).

**Table 3:** Effect of GEE on blood biochemistry in db/db mice. LDL: low-density lipoprotein; HbA1c: hemoglobin A1c.

	Group	Blood Biochemistry				
		db/+	db/db			
Parameters		GEE 0 *	GEE 0 *	GEE 0 *	GEE 50 *	GEE 200 *
		Metformin 0 *	Metformin 0 *	Metformin 140 *	Metformin 0 *	Metformin 0 *
Insulin		0.17 ± 0.08 ^b^	1.06 ± 0.54 ^a^	0.52 ± 0.20 ^ab^	0.91 ± 0.20 ^a^	0.58 ± 0.30 ^ab^
C-peptide		35.7 ± 25.8 ^b^	336.0 ± 168.0 ^a^	178.2 ± 97.3 ^ab^	159.3 ± 167.4 ^b^	145.2 ± 57.0 ^b^
HbA1c (ng/mL)		14.6 ± 2.5 ^b^	28.0 ± 4.1 ^a^	19.7 ± 1.9 ^bc^	21.7 ± 2.0 ^b^	16.1 ± 4.2 ^cd^
LDL (mg/dL)		14.7 ± 0.3	14.4 ± 1.0	14.5 ± 0.9	14.4 ± 1.4	14.1 ± 0.9
Triglyceride (mg/dL)		75.3 ± 8.0 ^b^	341.5 ± 97.2 ^a^	282.2 ± 108.5 ^a^	237.5 ± 136.9 ^a^	315.8 ± 174.0 ^a^
T. cholesterol (mg/dL)		95.1 ± 9.1 ^b^	177.8 ± 24.0 ^a^	156.7 ± 21.9 ^a^	157.8 ± 33.5 ^a^	152.7 ± 15.6 ^a^

* (mg/kg/day), (*n* = 8 per group, ^a^ *p* < 0.05, ^b^ *p* < 0.01, ^c^ *p* < 0.001).
